# Why Physicians Cannot Consult with Homœopaths

**Published:** 1885-12

**Authors:** 


### Medical ★ Journal,

PUBLISHED MONTHLY AT

AUSTIN, TEXAS.

F. E. DANIEL, M. D., Editor and Publisher

Subscription Price: Two Dollars per Annum in Advance.

Papers for publication in this Journal should be in the hands of the Editor by the 10th of the month preceding the month in which it is wished to appear; they must be original,—never having appeared in print elsewhere, and must be contributed exclusively to this Journal.

Contributions to the department of Original Articles are cordially invited from the professiom of Texas, expecially. What is most desired is Clinical reports of cases, essays and short practical articles on any medical topic; also solicited reports of proceedings of societies, clubs, etc., and items of medical news of general interest to the profession, such as notices of appointments of physicians, removals, changes, marriages, deaths, etc.

### Editorial.

### WHY PHYSICIANS CANNOT CONSULT WITH HOMŒOPATHS.

The average specimen of the *genus* homœopath poses as a martyr, and essays the *role* of "injured innocence." The complaint is often made by them that "old school physicians refuse to consult with us, as if homœopathy were a crime."

The laity do not know the difference between the practice of the two "schools," as they are called, and attribute the refusal of physicians to affiliate with homœopaths to jealousy, or something of the kind. They do not know the impossibility of a consultation, such as is referred to; they suppose there is mere a technicality which should be sunk in the interest of humanity. We propose, briefly, to explain why it is that a regular physician *cannot* associate with a homœopath in the management of a case, nor even give his aid in consultation.

We will take the affirmative in the above proposition, and will endeavor to show, first, that homœopathy *is* a crime.

There are crimes of omission and crimes of commission.

If a child is suffering with acute dysentery, for illustration, to give that child aloes or arsenic, powerful irritants to the alimentary mucous membrane, and which, in overdoses, produce dysenteric symptoms, straining, bloody passages, and death, can, with justice, be called a crime of commission; or, to give that child simples, or *sugar of milk* at the crisis of danger—to the neglect of remedies known to have a curative influence over diseased mucous membrane, at a time when the tenesmus is constant and distressing, the stools, or attempts at stool, continuous, until the child is literally exhausted, and dies in agony, might, in charity, be called a crime of omission; but as it is known, and is daily demonstrated that the pain in dysentery can, at least, be relieved, such practice assumes very much the aspect of criminality; it is inhuman.

We have the assurance of a most estimable and reliable lady that a child in this city, recently, under homœopathy treatment, had stools or attempts at stool continuously, suffering all the while, and died, finally, from sheer exhaustion. The medicine (?) she said, having not the slightest effect in either checking the tenesmus or relieving the pain; there was no fever.

The daily papers announced the death of General McClellan in blazing headlines, thus. "General McClellan expires in the agony of neuralgia of the heart in the presence of a homœopath who is powerless to relieve his sufferings;" and in commenting on his death, the paper said: "The remedies suggested by the skill of the physician were of no more avail than those applied by the wife and daughter of the patient."

What a commentary on homœopathy! A hypodermic injection of morphia and atropia—at once, powerful heart stimulant—and *sedative*, would, perhaps, have saved that valuable life. Not to have given him the benefit of all the resources known to medical and surgical science, amounts, in our judgment, to a crime, both of omission and commission. If euthanasia were ever justified, it would be in a case of that character, supposing the disease to have been beyond human skill to cure; humanity demands that the pangs be mitigated, at least, and the man allowed to die in peace.

We do not wish to be understood as admitting, by any means, that *because* homœopathy is a sin, physicians refuse to consult with homœopaths; but these persons want to make it appear that they are *persecuted*, and that there is *no* valid reason why we should refuse them counsel; that it is done out of ill-will, bigotry, etc. That is their game to make sympathy, and to get newspaper advertising gratuitously. None know better than they do the true reasons of refusal, and it is to gain popular sympathy that they persist in requesting it, in the face of repeated refusals.

Persons, who argue from opposite premises, can never reach the same conclusions. Homœopathy professes to believe that "like cures like." Physicians know that most frequently, unlike cures like, if we may be permitted such an expression to convey the idea of dis-similars. Homœopathy teaches, or professes to believe that the effects of a medicine bear an inverse ratio to its strength, or, rather, that the strength of the medicine bears an inverse ratio to the size of the dose,—the smaller the dose, the stronger the medicine! And we have the authority of the august one-hundred-pound President of the august thirty-five strong Homœopathic State Association, or State Homœopathic Association, we don't know which they call it, for saying "the origin of homœopathy was the discovery (?) by Hahneman that peruvian bark, taken in health, will produce a chill!" How absurd! If there is peruvian bark enough in Texas, or rather, little enough, to give us a chill, and it does so, we will turn homœopath and try to sprout side whiskers and look wise. What was possible in Hahneman's day should be possible now, as he flourished long after the *other* apostles, and after the days of miracles had ceased; and what was possible with Hahneman ought to be possible with any other man.

Now, we ask, in view of the foregoing *professions* of so called principles which, it is claimed, constitute the foundation of the homœopathic system, so called, how can a regularly educated physician—one who has been thoroughly grounded in the grand truths of the science of medicine, with any hope of benefiting the patient, and without sacrificing his self respect, hold a consultation with a person who practices, or pretends to practice, in accordance with a doctrine so diametrically opposed to our every-day experience, reason and common sense?

For the sake of illustrating the absurdity of the thing, we will suppose that a physician, forgetting his self respect and the principles of the physicians' code of ethics, that anchor and compass, which is at once his foundation and guide, is induced, out of consideration for the welfare of some patient, to hold a consultation with a homœopath. It is a case of acute dysentery, under homœopathic treatment. The child has fever; inflammation of the rectum, great tenesmus, frequent small bloody stools of mucus and epithelial casts, with small, weak pulse. The physician inquires "what are you giving Doctor?" Answer—"Arsenicum." (or "aloes"), in all probability, as, according to the homœopathic doctrine, these drugs—known to irritate and inflame the bowels and to produce bloody stools—would be "indicated." The physician naturally objects to the treatment, and proposes the opposite—emolient, *anodyne* remedies, which are as objectionable to the homœopath, as his course is to the physician. Thus the homœopath cannot accept his advice without a *total abandonment of his whole theory of treatment and an acknowledgement of its fallacy in conception and practice;* and there is the point, and difficulty. A physician cannot consult with a homœopath without either abandoning *his* wiews of therapeutics and practice, or without asking and expecting the homœopath to give up his; and no man of sensibility can consistently ask another to do what he cannot himself conscientiously do; and for that reason, and many other, consultations are necessarily declined. This reason has moreover, repeatedly been assigned; they know why it is; and understand the utter futility of expecting to reach common ground, without a total surrender of principles on one side or the other.

Prof. Frank Hastings Hamilton, in his excellent little work "Medical Ethics," illustrates, most appropriately the absurdity of holding a consultation with one of the homœopathic pursuasion. He makes an application of similar doctrines (to those of Hahneman) to the practice of law. He supposes a sect of German lawyers which he calls the "Slyhoovens," and shows the absurdity of a combination or consultation between one of them and a "regular" lawyer as follows :

*Counsel to the Judge—*

"Your Honor, I wish to say a word in reference to Mr. Kraft, my associated counsel in this case; as the conditions of his employment and his mode of practice are peculiar, and perhaps unusual; yet my client insists that he shall take part in the conduct of his defense. Mr. Kraft is a regularly authorized practitioner, according to the laws of this State; but he is a "Slyhooven" lawyer. He belongs to the "Slyhooven" school?"

*The Judge:*—"What is a Slyhooven lawyer, sir ?"

*Counsel :*—"A Slyhooven lawyer, please your Honor, is one who adopts the principles of *Slyhooven*, a celebrated German jurist, who holds that *the less the evidence, the stronger the proof*, and that if you can make the evidence *infinitesimal* you are certain to persuade the jury, and to win the case; provided, however, that in all cases you use that kind of evidence *which does not antagonize or contradict the evidence presented on the other side*" [the symptoms of disease.] "The hair of the same dog cures," is Mr. Slyhooven's cardinal maxim.

"Thus, for example, please your Honor, my client, being accused of stealing a horse, Mr. Kraft will present to the court the smallest possible amount of evidence to show that he *did actually steal a horse*,—the less the better for our client. Or, if no testimony can be found that he stole a horse, we shall avail ourselves of the next best form of testimony, namely, that he stole something closely resembling a horse. Now a mule resembles a horse, but it is not a horse. So we will present an infinitesimal fraction of testimony to prove that he stole a mule. If we can do that, we can acquit our client."

The allegory is carried further, and shows that neither the counsel nor the disciple of Slyhooven *really believed* such stuff, but he makes the jury and the client (homœopathic people) believe it, all the same.

And this is another point : these so called homœopaths don't *believe* what they profess; nor do they really practice it in all cases. Therefore, in addition to their being sinners by omission and commission, they are hypocrites besides. When they do so practice they generally lose their patient, as in General McClelland's case, and the one instanced above.

On the contrary, when they encounter a grave case—one, the tendency of which is to fatal termination, if they recognize its gravity, they quickly abandon their simples. But too often they are ignorant of the true nature of the malady, and *do nothing* till it is too late, their diagnostic powers, often, being insufficient, as they know nor care for, neither etiology nor pathology—symptoms only; they treat symptoms. They do know, however, that the tendency of many diseases is to recovery—we may say—the majority of ordinary diseases—and that rest and abstinence from food, will suffice in many cases; if left alone to that *vis medicatrix naturæ* which is often superior to the skill of the physician, they will recover; and hence their boasted successes; for, the pellet system—"little drops of water and little grains of milk"—as the *New York Medical Record*, a *quasi* homœopathic sympathizer pathetically sings, is tantamount to doing nothing at all.

But when they do encounter and recognize a deadly disease—one wherein the skill of the physician—an intimate knowledge of disease—a profound acquaintance with pathology and the effects of drugs would avail—would come like the relief-laden camels at Lucknow, to the rescue of an overtaxed nervous system, and enable it to throw off the morbific influences as did the besieged the pressing hosts; or to neutralize and then eliminate them from the system,—hear them cry out for consultation with a "regular"; or, in despair, abandon the frail reed of homœopathy, as advised by the Patriarch of New Orleans and the South;—or—let the patient die!—and then *whine* in the *newspapers* because "refused counsel" by a despised, "bigoted regular."

The advice alluded to above, came,—not from the lips of "babies and sucklings"; not from a piping tyro;—but from the *Mikado* of Hahnemandom,—the homœopathic *Mogul*;—the infinitesimal hard-hitter from *away back*—W. H. Holcomb of New Orleans, perhaps the most reputable, and influential homœopath in the South.

In an article recently published in one of their little windy organs, (and which by the bye the *N. O. Medical and Surgical*

*Journal* treats to a "benefit," worth, one of our Austin physicians says—"a year's subscription"), this Father in Lilliputian Medicine, says:

"But you must not hesitate to avail yourself, if necessary, of active counter irritation, dry cupping, painting with iodine, frictions with croton oil. *I am now speaking for the welfare of the patient and not for the conservatism of theoretic homœopathy,*" *W. H. Holcomb of New Orleans.*

We once heard of an old minister who said that when his horse ran away with him in his buggy, he "trusted to Providence till the breeching broke, then he jumped." This is what Holcomb advises his deluded followers to do; for he is looked up to as Hahneman's apostle and successor,—his vice-*Regent*, on whom the sacred mantle has fallen, by common consent and most of them follow his advice—trust to Hahnemanism till the breeching gives way, then they cry aloud for "a regular!—a regular! my Hahnemandom for a regular!"—or lose the patient.

Oh, ye (people) of wondrous faith! How long will ye be fed on moonshine, while "fast ebbs the life blood away?"—listening to the syren song of "sugar pills—and no drug bills:" lured by the Lorelei's kiss, and forgetting the last chance for life—go down to the grave, blinded by an infatuation, and *for want of medical treatment.*

